# Computer Assisted Navigation Does Not Improve Outcomes in Posterior Fusion for Adolescent Idiopathic Scoliosis

**DOI:** 10.1177/21925682241274373

**Published:** 2024-08-08

**Authors:** Aaron Singh, Travis Kotzur, Blaire Peterson, Ezekial Koslosky, Chimobi Emukah, Christopher Chaput

**Affiliations:** Department of Orthopaedic Surgery, 14742 UT Health San Antonio, San Antonio, TX, USA

**Keywords:** orthopedics, spine surgery, navigation, scoliosis, posterior fusion, cost analysis, complications

## Abstract

**Study Design:**

Retrospective Cohort Study.

**Objective:**

The aim of this study was to compare the efficacy of CT-based computer assisted navigation (CAN) to conventional pedicle screw placement for patients with Adolescent Idiopathic Scoliosis (AIS).

**Methods:**

This retrospective cohort study drew data from the National Readmissions Database, years 2016-2019. Patients undergoing posterior fusion for AIS, either via CAN or fluoroscopic-guided procedures, were identified via ICD-10 codes. Multivariate regression was performed to compare outcomes between operative techniques. Negative binomial regression was used to asses discharge disposition, while Gamma regression was performed to assess length of stay (LOS) and total charges. Patient demographics and comorbidities, measured via the Elixhauser comorbidity index, were both controlled for in our regression analysis.

**Results:**

28,868 patients, 2095 (7.3%) undergoing a CAN procedure, were included in our analysis. Patients undergoing CAN procedures had increased surgical complications (Odds Ratio (OR) 2.23; *P* < 0.001), namely, blood transfusions (OR 2.47; *P* < 0.001). Discharge disposition and LOS were similar, as were reoperation and readmission rates; however, total charges were significantly greater in the CAN group (OR 1.37; *P* < 0.001). Mean charges were 191,489.42 (119,302.30) USD for conventional surgery vs 268 589.86 (105,636.78) USD for the CAN cohort.

**Conclusion:**

CAN in posterior fusion for AIS does not appear to decrease postoperative complications and is associated with an increased need for blood transfusions. Given the much higher total cost of care that was also seen with CAN, this study calls into question whether the use of CAN is justified in this setting.

## Introduction

Adolescent Idiopathic Scoliosis (AIS) is a three-dimensional deformity of the spine that affects up to 4% of adolescents aged 10 until skeletal maturity.^1,2^ While the majority of cases are mild and are amenable to conservative treatment, typically bracing, more advanced disease requires surgical intervention, as severe disease risks respiratory compromise and worsening deformity. Even less severe disease has been associated with pain, cosmetic concerns, and psychosocial issues.^1,3-8^ Most commonly, operative management of AIS is posterior instrumentation and fusion of the involved segments.^9^

While surgery is generally successful at correcting and stopping the progression of deformity, complications, particularly those associated with pedicle screw malposition, are still cause for concern.^10^ Conventional free-hand or fluoroscopic guided pedicle screw placement, while commonly performed, results in high rates of malpositioned screws, with reports in the literature ranging from 5-41% in the lumbar spine and 3-55% in the thoracic spine.^11^ With vital neurovascular structures in the surrounding area, avoiding violation of the spinal canal and walls of the pedicle are key concerns during screw placement.^12^

Indeed, in recent years, the use of navigation techniques has promised to improve pedicle screw placement,^13,14^ and theoretically reduce screw related complications.^15^ Despite these promising results, the literature is equivocal regarding clinically significant improvements in outcomes, and it is likely that the efficacy of these navigation techniques vary based on procedure, specific type of navigation, and level.^15-24^ Still, Kelley et al found that computer assisted navigation (CAN) was utilized in just 0.04% of spinal fusion surgeries in 2004, but had increased to 3.3% by 2014; however, they noted that further research was still needed to assess the impact of this technology on clinical outcomes.^25^

The aim of this study was to assess the impact of CAN on clinical outcomes and cost in the setting of posterior fusion surgery for AIS. We hypothesized that CAN would reduce complications and improve overall clinical outcomes.

## Materials and Methods

### Data Source, Study Design, and Collection

Informed consent was not required for this study. This retrospective cohort study assessed the impact of CAN on clinical outcomes and cost following posterior fusion surgery for AIS, drawing data from the National Readmissions Database (NRD), years 2016-2019. The NRD is a national database with discharge and readmissions data from 31 states, representative of 62.2 percent of the U.S. population, and up to 32 million discharges, or 60.8 percent of all hospitalizations. The NRD links patient level discharge and hospitalization records within a calendar year, allowing assessment of patients, and a variety of outcomes, over time.

With the database, International Classification of Diseases, Tenth Revision, Clinical Modification/Procedure Coding System (ICD-10) codes were utilized to identify patients with AIS undergoing posterior fusion. Then, those whose procedure was assisted by CAN were identified to serve as the CAN cohort, while those who underwent the operation without CAN represented the conventional cohort. Importantly, patients in the conventional cohort underwent conventional free-hand or fluoroscopic assisted procedures, and those with other forms of navigation or assistance, such as robotic screw placement, were excluded. As the NRD follows patients for one calendar year, only patients who underwent surgery from January 1st through November 30th were included to ensure the ability to assess outcomes for a minimum of 30-days postoperatively. No Institutional Review Board (IRB) approval was required for this study; the IRB exemption number is 20230793NRR.

A number of preoperative variables were collected, including patient demographics such as sex, age, household income level, and insurance status. Using ICD-10 codes, we also calculated an Elixhauser Comorbidity Index score for each patient.

With respect to outcomes, both postoperative complications and hospital associated outcomes, including cost, were assessed. Complications assessed included both medical complications (respiratory failure, pulmonary embolism, pneumonia, cardiac arrest, heart failure, myocardial infarction, deep vein thrombosis, acute kidney injury, urological infections, stroke, plegia and paresis, osteomyelitis, sepsis), surgical complications (mechanical failure, postoperative neurological complications, transfusion, postoperative vascular complications, and postoperative shock), and mortality. Hospital associated outcomes included 30-day readmission, 30-day reoperation, length of stay (LOS), discharge disposition, and total charges.

The use of an exceptionally large, national sample also allowed us to trend the utilization of CAN in posterior fusion surgery for AIS. We assess the total number and proportion of CAN procedures per year across the study period.

### Statistical Analysis

To ensure the validity of our analysis, we confirmed that the assumptions of a valid logistic and linear regression model were met. For logistic regression, we verified that the dependent variable was dichotomous, the absence of multicollinearity among predictor variables, and the linearity of the logit for each continuous independent variable. For linear regression, we assessed the normality of residuals, the independence of observations, the homoscedasticity of residuals, and the linearity of the relationship between each independent variable and the dependent variable.

Categorical results are reported as counts with column percentages. Continuous data are reported as means standard deviations; standard errors are given where appropriate. Comparison of normally distributed data was performed with independent sample t tests. For non-normally distributed data, the Wilcoxon rank-sum test was performed. Categorical variables were assessed with Fisher’s Exact Test or Chi Square with Kendall Tau. Where appropriate, residuals were assessed for normal distribution and no multicollinearity was observed.

For all regressions, where appropriate, patient demographics and comorbidity burden, measured via Elixhauser Comorbidity Index score, were controlled for. Multivariate regression was performed to assess postoperative complications. Negative binomial regression was performed to assess readmissions, reoperations, and discharge disposition. Gamma regression was performed to assess total charges and LOS. Confidence intervals were set to 95% and *P*-values of 0.05 or less were considered significant. All analysis was performed in R Foundation for Statistical Computing software version 4.20.

## Results

### Demographics

**Figure 1.**
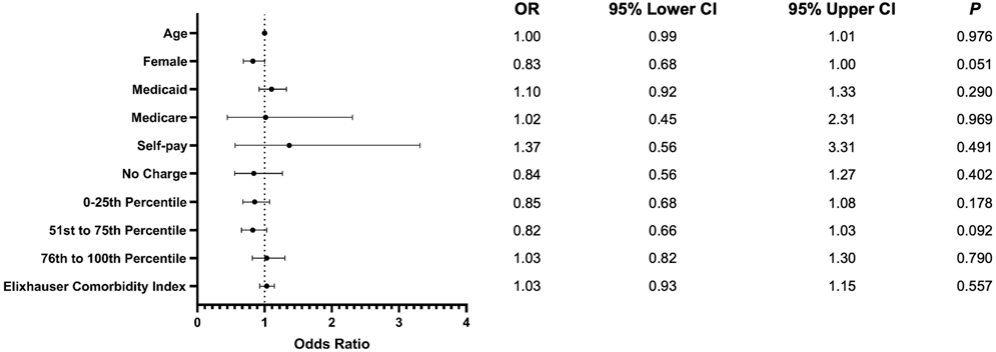
Forest plot comparing demographics. Odds of outcomes are given as computer assisted relative to conventional posterior fusion surgery.

A total of 28 868 patients undergoing posterior fusion for AIS were included in our analysis. 2095 (7.3%) underwent a procedure utilizing CAN. 22,062 (76%) of patients were female. 9711 (34%) had Medicaid, 17 341 (60%) had private insurance, and 310 (1.1%) had Medicare. Full demographics, with counts and proportions, can be seen in Table 1. The comorbidity burden, assessed via the Elixhauser Comorbidity Index, of both cohorts can be seen in Table 2. We found no significant differences in the demographics of patients undergoing conventional vs CAN procedures. These regression results can be seen, with 95% confidence intervals, in Figure 1.

**Table 1.** Demographics.

Demographics	Overall (N = 28,868)^a^	Conventional (N = 26,773)^a^	Computer Assisted Navigation (N = 2095)^a^	*P*-value^b^
Age	15.36 (6.85)	15.37 (6.92)	15.22 (5.84)	0.13
Gender				0.006
Female	22,062 (76%)	20,559 (77%)	1503 (72%)	
Male	6806 (24%)	6214 (23%)	592 (28%)	
Household income by zipcode				0.5
0-25th percentile	6845 (24%)	6321 (24%)	523 (25%)	
26th to 50th percentile	7527 (26%)	6933 (26%)	594 (28%)	
51st to 75th percentile	7582 (26%)	7104 (27%)	479 (23%)	
76th to 100th percentile	6914 (24%)	6415 (24%)	500 (24%)	
Payer				0.5
Medicaid	9711 (34%)	8991 (34%)	721 (34%)	
Medicare	310 (1.1%)	298 (1.1%)	13 (0.6%)	
Other	1255 (4.3%)	1182 (4.4%)	73 (3.5%)	
Private insurance	17,341 (60%)	16,080 (60%)	1261 (60%)	
Self-pay	251 (0.9%)	223 (0.8%)	28 (1.3%)	
Time to procedure	0.16 (1.48)	0.17 (1.53)	0.10 (0.62)	0.9
Length of stay	4.06 (2.78)	4.08 (2.86)	3.91 (1.47)	0.5
Total charge	197,085.84 (120,036.32)	191,489.42 (119,302.30)	268,589.86 (10,636.78)	<0.001
Discharge disposition				0.055
Adverse discharge	45 (0.2%)	41 (0.2%)	3 (0.2%)	
Home discharge with care	1113 (3.9%)	1075 (4.0%)	38 (1.8%)	
Routine discharge	27,463 (95%)	25 420 (95%)	2043 (98%)	
Transfer to skilled facility	247 (0.9%)	236 (0.9%)	11 (0.5%)	
Elixhauser comorbidity index	0.40 (0.77)	0.39 (0.77)	0.45 (0.77)	0.2
30-d readmission	21 (<0.1%)	21 (<0.1%)	0 (0%)	0.5
7-d readmission	5 (<0.1%)	5 (<0.1%)	0 (0%)	0.7
30-d reoperation	13 (<0.1%)	13 (<0.1%)	0 (0%)	0.6

^a^N (%); Mean (SD).

^b^chi-squared test with Rao & Scott’s second-order correction; Wilcoxon rank-sum test for complex survey samples.

**Table 2.** Elixhauser Comorbities by Cohort.

Characteristic	Overall, N = 28868^a^	Conventional, N = 26,773^a^	Computer Assisted Navigation, N = 2,095^a^	*P*-value^b^
Congestive heart failure	40 (0.1%)	38 (0.1%)	1 (<0.1%)	0.4
Cardiac arrhythmias	1405 (4.9%)	1291 (4.8%)	114 (5.4%)	0.7
Valvular disease	236 (0.8%)	208 (0.8%)	28 (1.3%)	0.12
Pulmonary circulation disorders	70 (0.2%)	69 (0.3%)	1 (<0.1%)	0.2
Peripheral vascular disorders	48 (0.2%)	48 (0.2%)	0 (0%)	0.4
Hypertension	38 (0.1%)	37 (0.1%)	1 (<0.1%)	0.4
Paralysis	71 (0.2%)	69 (0.3%)	2 (<0.1%)	0.3
Other neurologic disorders	407 (1.4%)	393 (1.5%)	14 (0.7%)	0.15
Chronic pulmonary disease	3497 (12%)	3151 (12%)	345 (16%)	0.018
Diabetes	50 (0.2%)	50 (0.2%)	0 (0%)	0.4
Hypothyroidism	330 (1.1%)	306 (1.1%)	24 (1.1%)	>0.9
Renal failure	56 (0.2%)	55 (0.2%)	1 (<0.1%)	0.2
Liver disease	38 (0.1%)	32 (0.1%)	6 (0.3%)	0.11
Peptic ulcer disease (excluding bleeding)	1 (<0.1%)	1 (<0.1%)	0 (0%)	0.8
AIDS/HIV	3 (<0.1%)	3 (<0.1%)	0 (0%)	0.7
Lymphoma	5 (<0.1%)	5 (<0.1%)	0 (0%)	0.6
Metastatic cancer	6 (<0.1%)	6 (<0.1%)	0 (0%)	0.7
Solid tumor without metastasis	9 (<0.1%)	2 (<0.1%)	7 (0.3%)	<0.001
Rheumatoid arthritis/collagen vascular diseases	136 (0.5%)	124 (0.5%)	12 (0.6%)	0.6
Coagulopathy	547 (1.9%)	509 (1.9%)	39 (1.8%)	>0.9
Obesity	1285 (4.5%)	1154 (4.3%)	131 (6.3%)	0.2
Weight loss	166 (0.6%)	157 (0.6%)	9 (0.4%)	0.7
Fluid and electrolyte disorders	1225 (4.2%)	1179 (4.4%)	45 (2.2%)	0.046
Blood loss anemia	138 (0.5%)	138 (0.5%)	0 (0%)	0.3
Deficiency anemias	138 (0.5%)	134 (0.5%)	4 (0.2%)	0.2
Alcohol abuse	14 (<0.1%)	12 (<0.1%)	1 (<0.1%)	0.7
Drug abuse	154 (0.5%)	124 (0.5%)	30 (1.4%)	0.077
Psychoses	36 (0.1%)	24 (<0.1%)	12 (0.6%)	0.009
Depression	1016 (3.5%)	926 (3.5%)	90 (4.3%)	0.4

^a^N (%).

^b^chi-squared test with Rao & Scott’s second-order correction.

### Postoperative Complications

**Figure 2.**
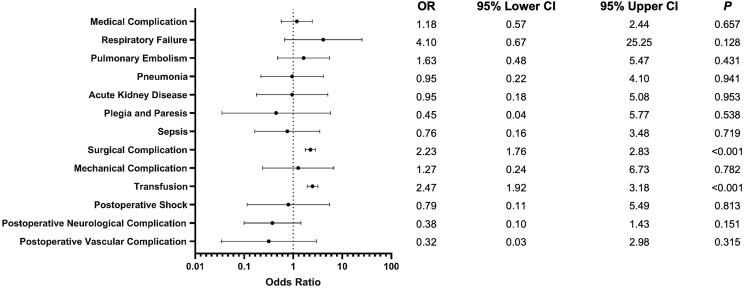
Forest plot comparing adverse events. Odds of outcomes are given as computer assisted relative to conventional posterior fusion surgery.

We found no difference in medical complications between CAN and conventional surgery; however, patients undergoing CAN had significantly higher rates of surgical complications (Odds Ratio (OR) 2.23; *P* < 0.001), including blood transfusions (OR 2.47; *P* < 0.001). There were no recorded instances of mortality within 30-days. Other complications were similar, with no significant difference observed, between groups. A breakdown of both medical and surgical complications, with counts and proportions, and univariate analysis results can be seen in Table 3. Multivariate regression results, including 95% confidence intervals, for complications can be seen in Figure 2.

**Table 3.** Medical and surgical complications by Cohort.

Characteristic	Overall, N = 28,868^a^	Conventional, N = 26,773^a^	Computer Assisted Navigation, N = 2,095^a^	*P*-value^b^
Medical complications	595 (2.1%)	533 (2.0%)	62 (2.9%)	0.4
Respiratory Failure	159 (0.6%)	153 (0.6%)	6 (0.3%)	0.2
Pulmonary embolism	43 (0.1%)	41 (0.2%)	1 (<0.1%)	0.4
Pneumonia	84 (0.3%)	76 (0.3%)	8 (0.4%)	0.7
Cardiac arrest	5 (<0.1%)	5 (<0.1%)	0 (0%)	0.7
Heart Failure	32 (0.1%)	30 (0.1%)	1 (<0.1%)	0.6
Myocardial infarction	4 (<0.1%)	3 (<0.1%)	1 (<0.1%)	0.13
Deep vein thrombosis	17 (<0.1%)	15 (<0.1%)	2 (<0.1%)	0.7
Acute kidney disease	80 (0.3%)	55 (0.2%)	25 (1.2%)	0.051
Urological infections	171 (0.6%)	152 (0.6%)	18 (0.9%)	0.3
Plegia and paresis	71 (0.2%)	69 (0.3%)	2 (<0.1%)	0.3
Sepsis	56 (0.2%)	50 (0.2%)	5 (0.3%)	0.6
Surgical	4972 (17%)	4101 (15%)	871 (42%)	0.001
Wound disruption	31 (0.1%)	31 (0.1%)	0 (0%)	0.4
Transfusion	4495 (16%)	3638 (14%)	857 (41%)	<0.001
Mechanical Failure	171 (0.6%)	166 (0.6%)	5 (0.2%)	0.077
Postop neuro	209 (0.7%)	198 (0.7%)	11 (0.5%)	0.6
Postop shock	128 (0.4%)	125 (0.5%)	4 (0.2%)	0.12
Postop vascular	93 (0.3%)	91 (0.3%)	2 (<0.1%)	0.10

^a^N (%).

^b^chi-squared test with Rao & Scott’s second-order correction.

### Hospital Related Outcomes

We found no significant difference in length of hospital stay or discharge disposition between the conventional and CAN cohorts. However, total charges were significantly greater in the CAN cohort (OR 1.37; *P* < 0.001). Mean charges were 191489.42 USD for conventional surgery vs 268 589.86 USD for the CAN cohort.

**Figure 3.**
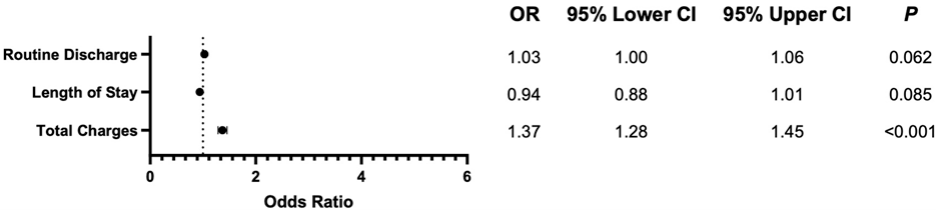
Forest plot comparing hospital outcomes. Odds of outcomes are given as computer assisted relative to conventional posterior fusion surgery.

For both 30-day readmission and 30-day reoperation rates, no significant differences were observed between the groups. The CAN group experienced no readmissions or reoperations within 30 days. In contrast, the conventional cohort reported 21 readmission events (<0.1%) and 13 reoperation events (<0.1%) within the same period. Full results for hospital associated outcomes can be seen in Figure 3.

### Utilization of CAN

**Figure 4.**
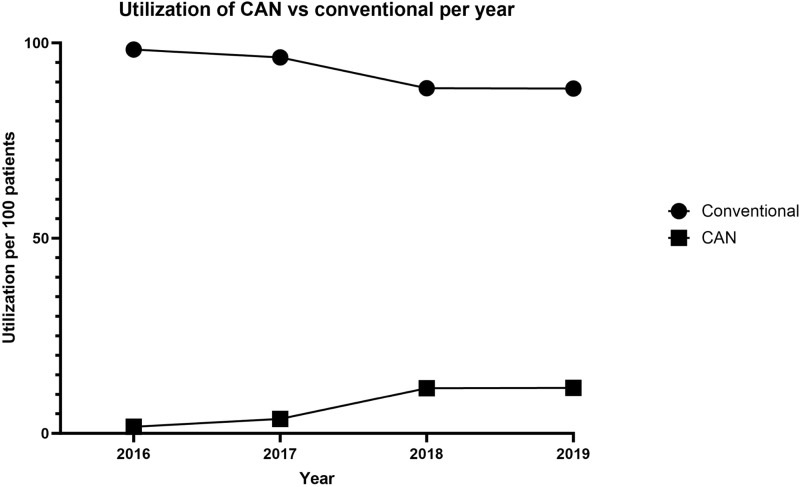
Utilization of computer-assisted navigation vs conventional surgery, per year.

The utilization of CAN in posterior fusion for AIS increased across the study period. In 2016, 118, or 1.7%, of procedures, were performed with CAN. In 2019, this rose to 884, or 11.7%, of procedures. Counts and proportions, by year, can be seen in Table 4 while trend lines in utilization for both conventional and CAN procedures can be seen in Figure 4.

**Table 4.** Computer Assisted Navigation Utilization by Year.

Year	Total	Conventional	Computer Assisted Navigation
2016	7088	6970 (98.3%)	118 (1.7%)
2017	7097	6832 (96.3%)	265 (3.7%)
2018	7138	6310 (88.4%)	827 (11.6%)
2019	7545	6660 (88.3%)	884 (11.7%)

## Discussion

This study assessed whether computer assisted navigation (CAN) improves outcomes for patients undergoing posterior fusion surgery for AIS compared to conventional procedures. Although CAN has repeatedly demonstrated superiority with respect to accuracy of pedicle screw placement, evidence of superior clinical outcomes in posterior fusion for AIS is lacking. We found that CAN did not improve outcomes; furthermore, our findings suggest it may increase complications. Controlling for other variables, CAN was 37% more expensive with 2.47 greater odds of requiring a transfusion. Our results suggest that CAN significantly increases costs and the need for transfusion, calling into question its use in posterior fusion for AIS.

The increased rates of blood transfusions are troubling, and likely suggest greater blood loss during CAN procedures. We hypothesize that this is due to increased operation times in CAN assisted procedures. Moore et al. reported that navigation, in the setting of posterior fusion for AIS, increased operative time by 41 minutes, with no difference in levels fused.^26^ Similarly, in the setting of lumbar fusion surgery, Nooh et al. found that computer assistance increased operation time by over 20 minutes.^27^ Prolonged operation time increases the risk of requiring a blood transfusion, and Tang et al. found that, in the setting of posterior fusion for AIS, for each additional minute of operation time, there was an increase of 1.5% in having significant blood loss (over 500 mL).^28^ It is unknown if selection bias could have also played a role in the difference (e.g. if CAN were preferentially used in more complex, larger cases).

Still, current literature consistently suggests that CAN results in more accurate pedicle screw placement than conventional techniques^29-31^; however, the clinical significance of this improvement is unclear. A study by Schulze et al. found that, in a cohort with a pedicle screw placement accuracy of only 80%, there was no displacement of the dura, compression of the spinal nerves, or relevant neurologic changes to any patients in the study.^32^ However, they noted that accuracy may vary based on surgeon experience. Similarly, a literature review by Gautschi et al. analyzed 39 articles with 46 patient groups and concluded that complications related to screw misplacement are incredibly rare and occur in less than 0.5% of cases.^33^

While there is much literature debating the utility of CAN for pedicle screw placement in general, there is very little published on the impact of navigation on outcomes following posterior fusion for AIS, particularly from more recent years. Our results indicate that there is no benefit to CAN with respect to clinical outcomes. These findings are consistent with related literature. A retrospective study by Kaur et al. found that, in patients undergoing fusion, including combined and anterior procedures, for AIS using CAN vs freehand, there was no difference in neurological complications or reoperation within 90 days or 2 years. There was, however, a higher risk for overall medical complications within 90 days in the conventional cohort, but it was noted that there could be selection bias for higher volume care hospitals in the CAN cohort affecting those results. Furthermore, there is a great concern for additional selection bias due to the follow up parameters. While they identified 12 046 patients undergoing spinal fusion for AIS, only 8640 had data sufficient for 90-day follow up, and only a little more than one-third (4,468), had the full two-year follow-up. Their navigated cohort was limited to just 467 patients, over 9 years of data, substantially smaller than our own. Also, our study drew data from as recently as 2019, vs 2015, an important consideration given the rapidly increasing utilization of CAN. Including only posterior fusion limits confounding that may result via assessing other related, but distinct procedures and approaches. Use of the anterior approach may also suggest worse curvature requiring a second approach, introducing additional bias. Furthermore, this study did not assess transfusion or bleeding risk, representing a substantial limitation. They did, however, have similar findings with respect to cost, reporting a $25,038 greater reimbursement for navigated procedures.

Our findings are further corroborated by Moore et al, who also found similar outcomes, with increased transfusions in navigated posterior fusion for AIS.^26^ Importantly, their analysis was conducted in the NSQIP, in which large, academic medical centers are heavily overrepresented. Their analysis was also limited to just 340 navigated cases over 7 years of data. Furthermore, they were unable to assess cost measures, and proposed further value-based investigation into the use of navigation technology in this setting.

In a meta-analysis of studies on procedures requiring pedicle screw placement, Verma et al. found that CAN did not result in significant improvements in postoperative outcomes.^34^ Laine et al. conducted a randomized controlled study of patients undergoing posterior thoracolumbar or lumbosacral pedicle screw instrumentation and found that, while CAN did have a higher pedicle screw insertion accuracy, there was no difference in the rate of neurological complications or in the early clinical outcome.^35^ Notably, this study was limited to just 41 patients in the CAN cohort. In contrast, our study included almost fifty times as many CAN patients. In the setting of posterior cervical fusion, Ansari et al^36^ assessed 12 578 patients, 689 receiving CAN, and found no significant difference in the rates of reoperation, readmission, or 30-day complications.^36^ Similarly, Tang et al, also studying posterior cervical fusion, found no significant difference in 30-day and 90-day readmission rates or length of stay.^19^ In the setting of posterior lumbar fusion, however, Bovonratwet et al. found that patients receiving CAN had a statistically significantly shorter mean length of stay than those with a conventional procedure.^37^ Still, the difference was a mere 0.2 days, a difference that, while statistically significant, may not have clinical consequence. Notably, they found no difference in 30-day readmission or reoperation, suggesting the benefit, if any, was minimal.

Complications resulting from pedicle screw malpositioning are rare, and most malpositioning does not have clinical consequence, explaining the lack of improved outcomes with CAN. For example, a systematic review by Hicks et al. evaluated 1666 patients undergoing pedicle screw fixation for scoliosis and found that, although about 15% of patients had malpositioned screws, only 12 required reoperation for it.^38^ They also found no serious neurological complications, no vascular injuries, only one pulmonary complication, and no injury of surrounding anatomic structures. Still, complications related to malpositioning, when they occur, can be severe. However, it does not appear that CAN reduces the rate of these complications, at least within 30-days.

Establishing a clinical benefit to utilizing CAN is paramount, as it is consistently more expensive than conventional methods and may increase a patient’s financial burden.^19,20,25,39^ With the number of spine surgeries utilizing CAN increasing,^39^ and the price tag of AIS treatment increasing as well,^9^ surgeons need to determine if the guidance and potential increase in screw placement accuracy is worth the increased cost, particularly in light of the increased risk of transfusions and related morbidity.

## Limitations

While this study benefits from an extremely large, nationally representative sample, it is not without limitations. In particular, it is possible that CAN was used on more complex or difficult cases, contributing to the cost difference. However, baseline demographics were similar, and it is unlikely to have accounted for the very large differences in cost seen between CAN and traditional surgeries. Additionally, this was a retrospective study, and further, prospective or randomized controlled trials would provide more compelling evidence. Furthermore, we only assessed a select few outcomes within 30-days of surgery. We were unable to assess long-term outcomes, patient reported outcomes, or functional outcomes, all of which may play an important role in surgical decision making and cost-benefit analysis. Importantly, we lacked data on disease severity, such as Cobb angle, or number of levels fused, an important limitation of this study; however, similar studies report no difference in levels fused.^26^ Likewise, we lacked data specific to screw malposition; however, if there were a clinically significant difference that resulted in re-admission or reoperation, this should have been seen in the data available. Still, this represents one of the largest studies on the topic, utilizing a nationally representative database. Future, prospective and randomized controlled trials are still warranted to provide stronger evidence, examining outcomes for a longer follow-up period, and provide definite guidance on the use of CAN.

## Conclusion

The use of computer assisted navigation in the setting of posterior fusion for adolescent idiopathic scoliosis does not decrease the rate of complications, but it increases the odds of blood transfusions and is associated with significantly greater costs. While CAN has been shown to improve the accuracy of pedicle screw placement, this study did not observe any evidence of improved clinical outcomes. These findings do not support the use of CAN in routine posterior instrumentation and fusion for AIS.
